# Myeloid CCN3 protects against aortic valve calcification

**DOI:** 10.1186/s12964-022-01020-0

**Published:** 2023-01-20

**Authors:** Peinan Tu, Qian Xu, Xianming Zhou, Nicolas Villa-Roel, Sandeep Kumar, Nianguo Dong, Hanjoong Jo, Caiwen Ou, Zhiyong Lin

**Affiliations:** 1 Cardiology Division, Emory University School of Medicine, 101 Woodruff Circle, Room 3004, Atlanta, GA 30322 USA; 2 Affiliated Dongguan Hospital Southern Medical University (Dongguan People’s Hospital), Dongguan, 523058 China; 3 Department of Cardiovascular Surgery, Xiangya Hospital of Central South University, Changsha, China; 4 Department of Cardiovascular Surgery, Union Hospital, Tongji Medical College, Huazhong University of Science and Technology, Wuhan, China; 5 Wallace H. Coulter Department of Biomedical Engineering, Emory University and Georgia Institute of Technology, Atlanta, GA USA

**Keywords:** Myeloid, Macrophage CCN3, Valvular calcification, BMP2

## Abstract

**Background:**

Cellular communication network factor 3 (CCN3) has been implicated in the regulation of osteoblast differentiation. However, it is not known if CCN3 can regulate valvular calcification. While macrophages have been shown to regulate valvular calcification, the molecular and cellular mechanisms of this process remain poorly understood. In the present study, we investigated the role of macrophage-derived CCN3 in the progression of calcific aortic valve disease.

**Methods:**

Myeloid-specific knockout of CCN3 (Mye-CCN3-KO) and control mice were subjected to a single tail intravenous injection of AAV encoding mutant mPCSK9 (rAAV8/D377Y-mPCSK9) to induce hyperlipidemia. AAV-injected mice were then fed a high fat diet for 40 weeks. At the conclusion of high fat diet feeding, tissues were harvested and subjected to histologic and pathologic analyses. In vitro, bone marrow-derived macrophages (BMDM) were obtained from Mye-CCN3-KO and control mice and the expression of bone morphogenic protein signaling related gene were verified via quantitative real-time PCR and Western blotting. The BMDM conditioned medium was cocultured with human valvular intersititial cells which was artificially induced calcification to test the effect of the conditioned medium via Western blotting and Alizarin red staining.

**Results:**

Echocardiography revealed that both male and female Mye-CCN3-KO mice displayed compromised aortic valvular function accompanied by exacerbated valve thickness and cardiac dysfunction. Histologically, Alizarin-Red staining revealed a marked increase in aortic valve calcification in Mye-CCN3-KO mice when compared to the controls. In vitro, CCN3 deficiency augmented BMP2 production and secretion from bone marrow-derived macrophages. In addition, human valvular interstitial cells cultured with conditioned media from CCN3-deficient BMDMs resulted in exaggerated pro-calcifying gene expression and the consequent calcification.

**Conclusion:**

Our data uncovered a novel role of myeloid CCN3 in the regulation of aortic valve calcification. Modulation of BMP2 production and secretion in macrophages might serve as a key mechanism for macrophage-derived CCN3’s anti-calcification function in the development of CAVD.

Video Abstract

**Supplementary Information:**

The online version contains supplementary material available at 10.1186/s12964-022-01020-0.

## Background

Calcific aortic valve disease (CAVD), the most prevalent form of heart valve disease, is characterized by the thickening of the aortic valve due to significant fibrosis and/or calcification. Previous studies underscore the importance of chronic inflammation and dyslipidemia in the pathogenesis of this disease [1–4]. Efforts over the last several decades have identified risk factors (hypertension, aging, diabetes, obesity) and have begun to characterize the cellular and molecular mechanisms important for cardiac valve homeostasis. Studies have also identified aortic valve interstitial cells (VICs) as a critical cell type responsible for valvular remodeling and calcium deposition [5]. Therefore, due to the central importance of the transdifferentiation of VICs into osteoblast-like cells in the development of CAVD, VICs have been the focal cell type for CAVD pathogenesis studies. Accumulating evidence, however, also points to the importance of crosstalk between valve endothelial cells (VECs), circulating and resident immune cells, and VICs in the health and disease of the cardiac valve [6]. The role of innate immune cells, however, notably macrophages, in the modulation of valvular calcification has been well documented [7]. The remarkable plasticity bestowed upon macrophages allows them to polarize in a diverse array of disease settings including CAVD, in which macrophage-derived proteinases, such as cathepsins or metalloproteinases (MMP-2 and MMP-9), and inflammatory factors like TNF-α, IL-1β, and IL-6, have been demonstrated to have important roles in promoting VIC transdifferentiation and assuming osteogenic properties [2, 8]. While the importance of macrophages in the modulation of aortic valve calcification has been well acknowledged, the molecular and cellular mechanisms of macrophage action in CAVD remain far from complete. Importantly, little is known about the macrophage-derived factors that are salutary in CAVD.

The cellular communication network (CCN) family of proteins are a group of secreted matricellular proteins that interact with the extracellular matrix (ECM), growth factors, cell surface integrins and other receptors to promote ECM-intracellular signaling [9]. Increasing evidence highlights the previously underappreciated importance of the CCNs in both normal and disease states [10]. These proteins exert important functions in diverse physiological processes, including the hematologic, osteogenic, neural, pulmonary, and immune systems, and in pathologic states such as fibrosis, obesity, cancer, and many inflammatory conditions [10]. In bone biology, CCNs have been implicated in physiological development, turnover, and regeneration. For example, CCN1 modulates mature osteoblast and osteocyte function to affect bone mass [11], CCN2 regulates and coordinates osteoblast differentiation in skeletal development [12] and CCN4 regulates osteogenesis by enhancing BMP2 (bone morphogenic protein-2) activity and is also important for bone turnover [13, 14]. With respect to CCN3, in vivo studies support the notion that CCN3 negatively regulates bone regeneration via mechanisms involving BMP2 [15]. It is well appreciated that overlapping molecular machineries exist for bone formation and valvular calcification. Indeed, one key step for the precipitation of valvular calcification is the differentiation of VICs into osteoblast-like cells [5, 6]. In light of this, we speculated that CCN3 might participate in the regulation of aortic valve calcification.

In the current study, we investigated the role of CCN3 in CAVD. Building on our reported in vivo evidence demonstrating the inhibitory function of myeloid CCN3 in atherosclerosis [16], we tested our hypothesis that myeloid CCN3 mitigates aortic valve calcification, thus a protective factor against CAVD. We provide evidence that deficiency of myeloid CCN3 exacerbates aortic valve dysfunction and calcification. Additional in vitro studies highlight that augmentation of BMP2 production from CCN3-ablated macrophages serves as an important mechanism for the observed VIC calcification in the context of CCN3 deficiency.

## Materials and methods

### Human aortic valves collection

Calcified human aortic valves were obtained immediately following valve replacement surgeries and Noncalcified human aortic valves were obtained from transplant recipient hearts at Emory University Hospital Midtown according to the IRB-approved study at Emory University as previously described [17, 18]. Patient characteristics as well as the detailed VIC purity characterizations were described in detail in previous publication [19]. Both calcified and noncalcified leaflets were collected in the same way. Briefly, the aortic valves were washed in ice-cold phosphate buffered saline immediately following harvesting, and cusps were individually snap-frozen in optimal cutting temperature (O.C.T.) compound (Tissue-Tek). Valves were then sectioned (8 μm) in the radial direction and stored at − 80 °C for further use of immunohistochemical staining studies.

### Animal models and experiments

Myeloid-specific knockout of CCN3 (Mye-CCN3-KO) in mice was achieved by breeding CCN3-floxed mice (generated in our lab) with a LysM-Cre strain (The Jackson Laboratory). Mye-CCN3-KO mice and control mice (LysM-Cre/Cre) were used in these studies. All control and mutant mice were derived on a pure C57BL/6 J background. Mice were housed under standard light conditions (12-h light/12-h dark cycle) and allowed ad libitum access to standard laboratory diet and water. To generate CAVD in mice, hyperlipidemia was induced by knocking down the LDLR (low density lipoprotein receptor) with a single tail intravenous injection of AAV encoding mutant mPCSK9 (rAAV8/D377Y-mPCSK9) at 8–10 weeks of age [20]. AAV-injected mice were then fed a high fat diet (HFD) for 40 weeks, at which time mice were anesthetized with isoflurane (2% in oxygen) for heart function analysis with tissues subsequently harvested for histologic and pathologic analyses (Additional file 1: Fig. S1A). For tissue collection, mice were euthanized by excess carbon dioxide administration. All animal experiments were performed with the approval the Institutional Animal Care and Use Committee at Emory University, which is certified by the American Association of Accreditation for Laboratory Animal Care. All procedures in this study conform to the NIH Guide for the Care and Use of Laboratory Animals.

### Echocardiography

Echocardiography was performed using a Vevo 3100 device (FUJIFILM VisualSonics). Spectral Doppler was used to measure aortic valvular systolic velocity and M-mode on the horizontal axis of the left heart ventricle was used to measure heart function [21].

### Isolation of bone marrow-derived macrophages (BMDMs)

All primary macrophages used in assays are BMDMs. In light of the similar aortic valve dysfunction phenotype seen in both male and female Mye-CCN3-KO, only male mice were used for cell isolation. Bone marrow cells were collected from the hind legs of Mye-CCN3-KO mice and controls following the protocol as described previously [22]. Cells were seeded onto untreated Petri dishes, cultured in Dulbecco's Modified Eagles medium (DMEM) supplemented with L929 conditioned medium to allow for BMDM differentiation. BMDM purity was verified by immunofluorescence for CD68 (Bio-Rad MCA1957GA) and flow cytometry for CD11b and F4/80.

### Cell culture

Primary BMDMs isolated from Mye-CCN3-KO or control mice were cultured in DMEM with 10% fetal bovine serum containing 4.5 mg/mL glucose, 1% penicillin/streptomycin, and 1% glutamine. Human Aortic Valve Interstitial Cells (AVICs) were isolated from donated hearts not suitable for transplant (LifeLink of Georgia) as previously described [23, 24]. AVICs were cultured in DMEM supplemented with 1.0 mg/ml glucose,10% fetal bovine serum, 1% penicillin/streptomycin, 2 ng/ml basic fibroblast growth factor 2 (FGF2, Prospecbio, CYT-218) and 10 units/ml Heparin (Sigma, H7405-1G) and seeded on gelatin-coated glass-based dishes when not plated for experiments. Passages 4–7 were used for vitro assays. All cells were kept in a humidified incubator with 5% CO_2_ at 37 °C.

### Generation of BMDM conditioned media

BMDMs were seeded at 8.0 × 10^6^ cells/10 cm dish. After culturing BMDMs in fresh DMEM for seven hours, the medium was collected and centrifuged at 800 g for 5 min to remove the cellular debris, the resultant supernatant (conditioned media) was immediately frozen at − 80 °C and used for AVIC culture studies involving the application of conditioned media. Conditioned media was thawed at 37 °C immediately prior to addition to AVICs and was replaced every three days throughout the course of the culture experiments.

### ELISA

Supernatants collected from BMDMs were concentrated with Amicon Ultra-0.5 Centrifugal Filter Units (EMD Millipore, UFC501024). BMP2 concentration was assayed using the BMP2 Quantikine ELISA Kit (R&D System, DBP200). Each sample was measured in duplicate.

### Calcium mineralization and alizarin-red staining

Primary human AVICs were cultured with osteogenic BMDM conditioned medium (supplemented with 50 μg/ml ascorbic acid, 10 mmol/L β-glycerophosphate, 4.0 μg/ml cholecalciferol and 8 mM CaCl_2_) for 21 days with the culture medium changed every three days. On day 21, cells were washed and fixed with 4% formaldehyde for 15 min at room temperature. A 2% fresh Alizarin-Red solution (pH 4.1–4.3) was added to the cells and incubated for 20 min at room temperature with gentle shaking as described previously [25]. The solution was discarded, and cells were washed before image capture. Alizarin red quantification was performed as described in previous studies [25–27]. Briefly, calcium deposits were visualized by the red mineral deposits observed and quantified with ImageJ software. Data was normalized to the control osteogenic conditioned medium (OCM) group. For the assessment of calcification on tissue sections, 2% fresh Alizarin Red solution (pH 4.1–4.3) was applied for 5 min as previously described [24]. The positive acreage on the aortic valves was obtained via automated measurement using ImageJ and normalized to the total valve leaflet area.

### Histology and immunofluorescence

Human aortic valve leaflets from donated hearts were frozen in OCT and sectioned at 8 μm thickness. Murine aortic roots containing valve leaflets were frozen in OCT and sectioned at a thickness of 5 μm. For each sample, a total of 5 sections were obtained at 100 μm intervals and used in the histological assessment. For immunofluorescence, slides were blocked with 4% BSA and 0.5% Triton-X 100 in PBS for 1 h, then primary antibody staining was performed in blocking solution overnight at 4 °C. Secondary antibody staining was performed for 1 h at room temperature prior to staining with DAPI for 10 min. Slides were mounted using ProLong™ Diamond Antifade Mounting media (Thermo Fisher Scientific P36965) and imaged at 20X magnification with the slide imager (Keyence BZx-800 series). For the quantification of fluorescence staining in murine sections, positive signal acreage on each aortic valve was obtained via automated measurement using ImageJ and normalized to the valve leaflet area. For human tissue sections, CCN3, CD68 and DAPI positive cells were counted manually. CD68 positive cells were normalized to DAPI positive cells; and CCN3 positive cells were normalized to CD68 positive cells.

### Cell lysis and western blot

Cells were treated as described and then harvested for total protein using RIPA buffer supplemented with protease and phosphatase inhibitors (Thermo scientific, VI307208). For the determination of total mature-BMP2, BMDMs derived from 6 days of differentiation were cultured for seven hours in fresh medium. Cells and supernatants were collected together for immunoblotting by adding 1% Nonidet-P40 supplemented with protease inhibitor and 2 mM dithiothreitol directly to the well as previously described [28]. Cells were scraped and lysed on ice for 30 min followed by centrifugation at 12,000*g* for 10 min. Laemmle buffer (6X) was then added to the samples, boiled and 15 to 20 μg of protein samples were loaded onto an 8% to 20% SDS-PAGE gradient gel (Genscript, M41215).

### Real-time quantitative PCR

Total RNA was extracted using an RNA-isolation Kit (Qiagen, 74106) and cDNA was produced using reverse transcription supermix (Bio-Rad, 1708841). Real-time quantitative polymerase chain reaction (RT-qPCR) for all targets was performed on a QuantStudio 6 Flex Real-Time PCR Systems using RT2 SYBR®Green Rox qPCR master mix Kit (Qiagen, 330521). The relative quantification of the target gene was analyzed using the 2^−ΔΔCt^ method. GAPDH was used as an internal control.

### Statistics

GraphPad Prism 8.0 was used for statistical tests. Data are expressed as mean ± SEM. Normal distribution was examined using the Shapiro–Wilk test and homogeneity of variance using F tests. The data that passed both tests were then subjected to parametric analysis. To compare the means of two single groups, an unpaired Student’s *t*-test was used. To compare the means of more than 2 variables, One-way ANOVA followed by Dunnett’s post-hoc test was performed. To compare 2 independent variables, Two-way ANOVA followed by the Sidak multiple comparison test was used. P ≤ 0.05 was considered statistically significant.

## Results

### Myeloid CCN3 deficiency aggravates valvular dysfunction in mice

**Fig. 1:**
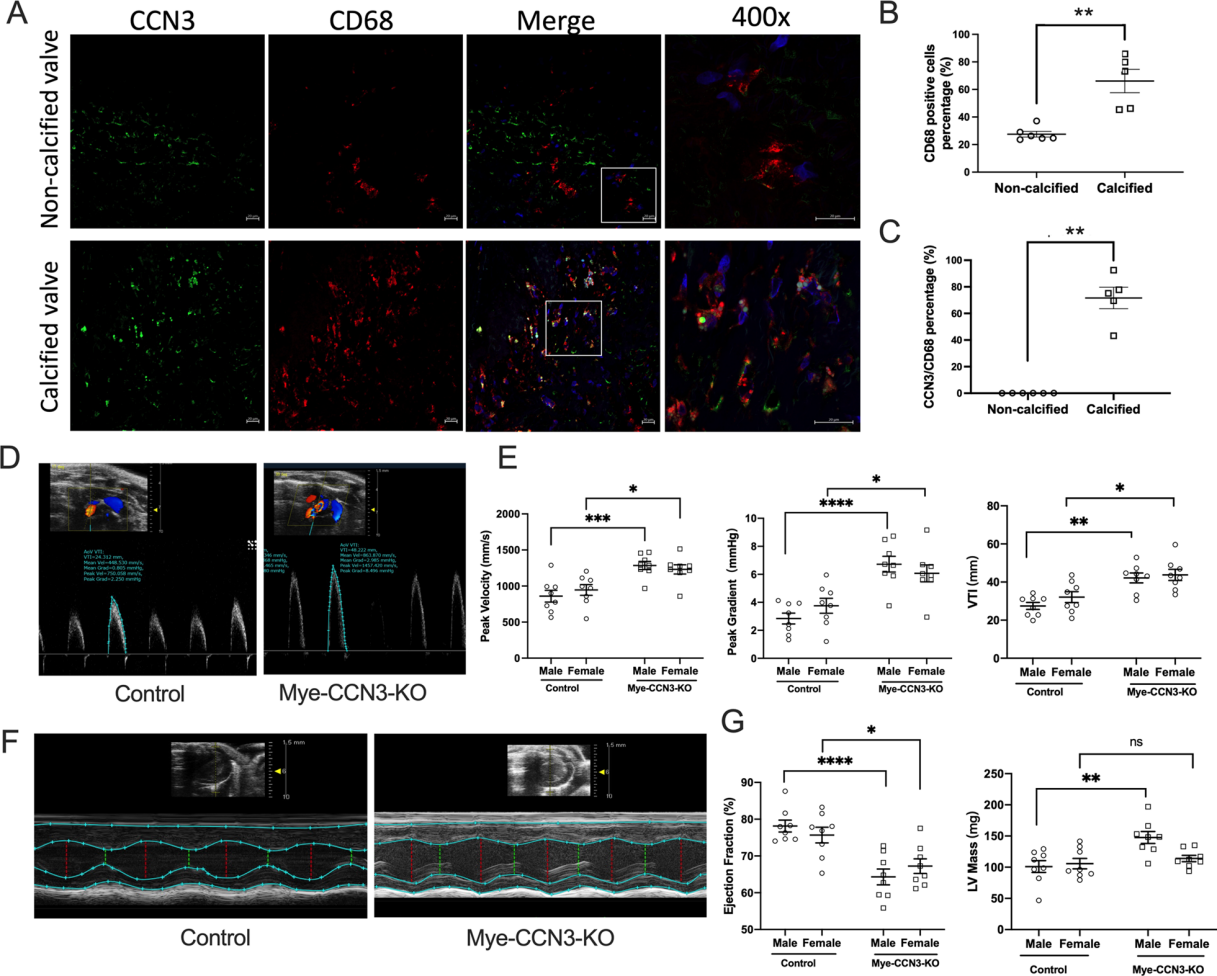
Myeloid deficiency of CCN3 induces aortic valvular dysfunction. **A** Representative immunofluorescence images of CCN3 and CD68 on either normal or calcified patient aortic valves. (CCN3, green; CD68, red; DAPI, blue; scale bar: 20 μm) **B** and **C** Quantification of CD68 expression and CCN3-CD68 co-expression from human non-calcific and calcific aortic valves. n = 6. **D** Representative images showing aortic valvular systolic velocity measurement by Spectral Doppler in mice. **E** Echocardiography results of peak velocity, peak gradient and VTI as quantified from doppler images (n = 8). **F** Representative M-mode echocardiography evaluation of murine cardiac function. **G** LV mass, ejection fraction and fraction shortening from M-mode echocardiography. All Statistical analysis were two-way ANOVA followed by Sidak multiple comparison test. N = biological replicates. Control: Lysm^Cre/Cre^ mice; Mye-CCN3-KO: Myeloid specific CCN3 knock out mice; VTI: velocity time integral; LV mass: left ventricle mass.*p < 0.05.**p < 0.01.***p < 0.001.****p < 0.0001

Cells of the myeloid lineage, including monocytes, macrophages, dendritic cells, and neutrophils, play key roles in the etiology of cardiovascular disease. Our previous studies have implicated a protective role of myeloid CCN3 against atherosclerosis and non-alcoholic fatty liver disease (NAFLD) [16, 29]. To gain initial insights into the role of myeloid CCN3 in CAVD, we assessed CCN3 protein levels in human CAVD. Our immunostaining of human valves revealed that, in comparison to non-calcified human valves, a significant increase (~ twofold) of CD68^+^ macrophages is seen in CAVD patients (Fig. 1A, B). Notably, co-staining studies demonstrated a strong increase of CCN3 in CD68^+^ macrophages on aortic valves from CAVD patients (Fig. 1A–C). On the basis of these findings, coupled with the established importance of innate immunity in a myriad of cardiovascular diseases, including CAVD, we speculated that myeloid CCN3 may affect the development of CAVD.

To test this hypothesis, we assessed the impact of myeloid CCN3 loss on aortic valve pathology in the context of hypercholesterolemia in conjunction with high fat diet feeding. Following the injection of AAV encoding mutant mPCSK9 (rAAV8/D377Y-mPCSK9), both Mye-CCN3-KO mice and controls were subjected to 40 weeks of HFD feeding (Additional file 1: Fig. S1A), at which time all study mice underwent echocardiography prior to tissue collection.

We analyzed peak transaortic velocity, peak gradient, and velocity time integral (VTI), all key parameters for the assessment of valvular stenosis. Interestingly and in line with our hypothesis, Mye-CCN3-KO mice exhibit dramatic aortic valve dysfunction when compared to control mice. As shown in Fig. 1D, E, based on Spectral Doppler analysis, peak velocity, peak gradient, and VTI were all significantly elevated in Mye-CCN3-KO mice irrespective of the sex, indicating that myeloid CCN3 deficiency leads to a hemodynamically significant aortic stenosis.

Aortic valve stenosis restricts blood flow from the left ventricle to the aorta, which with time leads to cardiac dysfunction. In our studies, M-mode echocardiography was performed to examine the left ventricle (LV) function. Our data revealed that, concordant with aortic valve dysfunction, a significant decrease of ejection fraction (EF) was seen in both Mye-CCN3-KO male and female mice (Fig. 1F, G), suggesting an occurrence of cardiac dysfunction. However, the LV mass changes showed a sex-dependent response where we noted a significant increase in the LV mass in males but not in females (Fig. 1G).

We also performed continuous wave Doppler to determine the presence of aortic valve regurgitation, which was reported in Wave-2 (EGFR kinase-deficient) mice [30]. Out of all the examined mice (8 males and 8 females per genotype), only two male Mye-CCN3-KO animals displayed severe regurgitation, while mild regurgitation was seen in two control mice and three Mye-CCN3-KO mice (Additional file 1: Fig. S1B and Fig. 1C). Our data suggests that the Mye-CCN3-KO mice develop aortic valve stenosis accompanied with low incidence of aortic regurgitation.

### Myeloid CCN3 deficiency exacerbates valvular calcification development

**Fig. 2:**
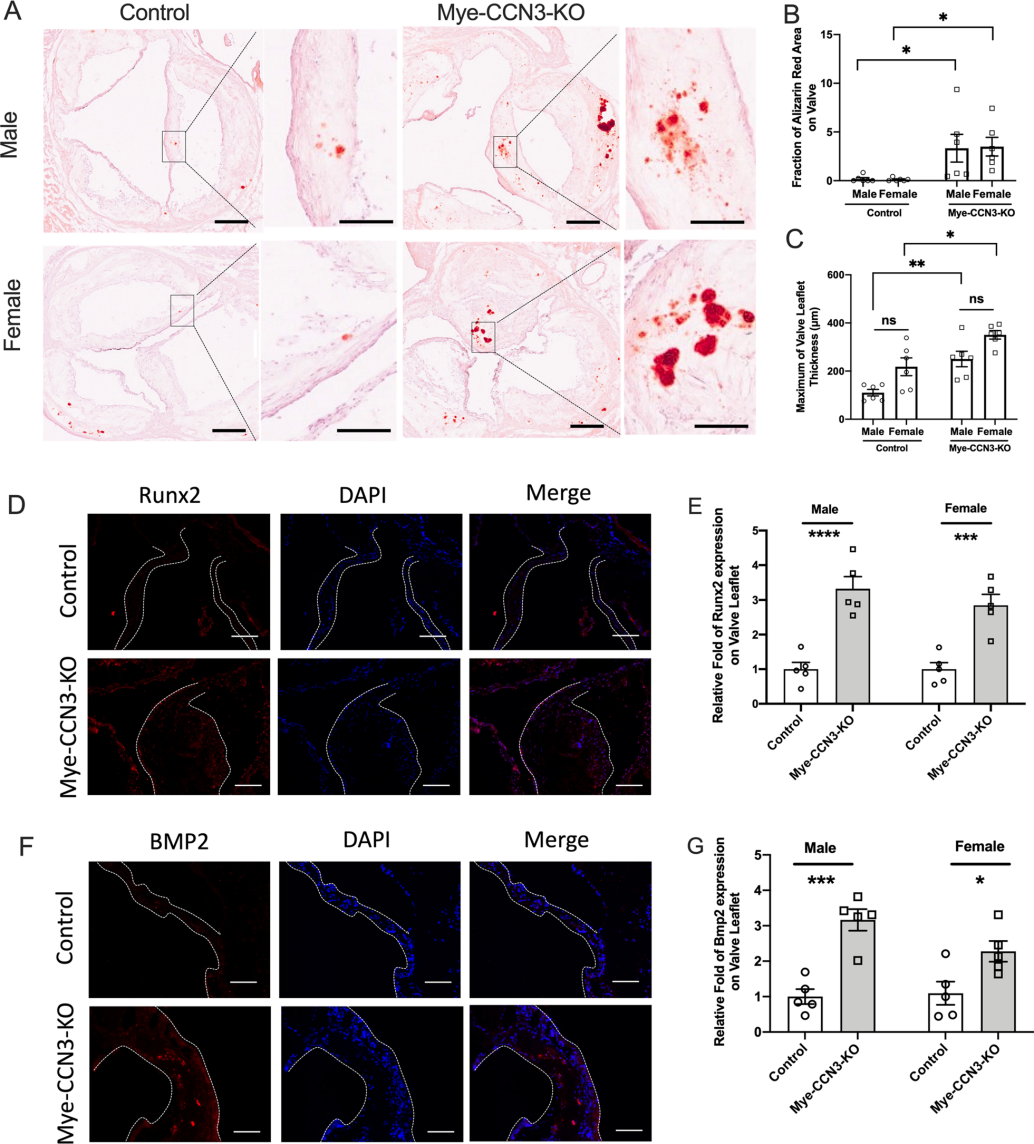
Myeloid CCN3 deficiency augments valvular calcification. **A** Representative Alizarin red staining images for valvular calcification. Scale bar: 200 μm. **B** and **C** Alizarin red positive area ratio to each valvular acreage (%) and maximum of valve leaflet thickness on mouse aortic valves were analysed by ImageJ (n = 6). **D** Immunofluorescence of Runx2 on calcified aortic valves. (Runx2: red; DAPI, blue; scale bar: 100 μm) **F** Immunofluorescence of BMP2 on calcified aortic valves. (BMP2: red; DAPI, blue; scale bar: 100 μm). **E** and **G** Quantification of the relative fold change of positive stained area to the entire valve leaflet area in comprision to the control group (n = 5). All Statistical analysis were two-way ANOVA followed by Sidak multiple comparison test. N = biological replicates. Control: Lysm^Cre/Cre^ mice; Mye-CCN3-KO: Myeloid specific CCN3 knock out mice. *p < 0.05.**p < 0.01.***p < 0.001.****p < 0.0001

Aortic stenosis has been long considered to occur as a result of progressive calcium buildup within the valve leaflets [31]. To assess the severity of valvular calcification in Mye-CCN3-KO mice, we stained Alizarin red on aortic valves to identify the carbonate minerals (Fig. 2A, B). In both male and female mice, Alizarin-Red staining revealed significantly elevated calcification in Mye-CCN3-KO mice. Concordantly, the maximum valve thickness is also significantly larger in the Mye-CCN3-KO group when compared to the control group (Fig. 2C), strongly supporting that calcification on the aortic valve leaflet is causal for the observed increased CAVD-like symptoms in the myeloid-CCN3-deficient state. Interestingly, in this current study, deficiency of myeloid CCN3 did not alter total cholesterol, high-density lipoprotein low-density lipoprotein, and triglycerides (Additional file 1: Fig. S2).

Consistent with the above observations, the expression of Runx2 (a central pro-osteogenic transcription factor) and BMP2 (a major signaling protein required for aortic valve calcification) [32], are significantly elevated on the aortic valve leaflets in Mye-CCN3-KO mice compared to controls irrespective of the sex (Fig. 2D–G).

Taken together, our data demonstrate that CCN3 deficiency in the myeloid compartment promotes murine CAVD, supporting a novel importance of myeloid CCN3 against valvular calcification.

### Myeloid CCN3 deficiency provokes BMP2 accumulation in VICs and macrophage recruitment on the aortic valve

**Fig. 3:**
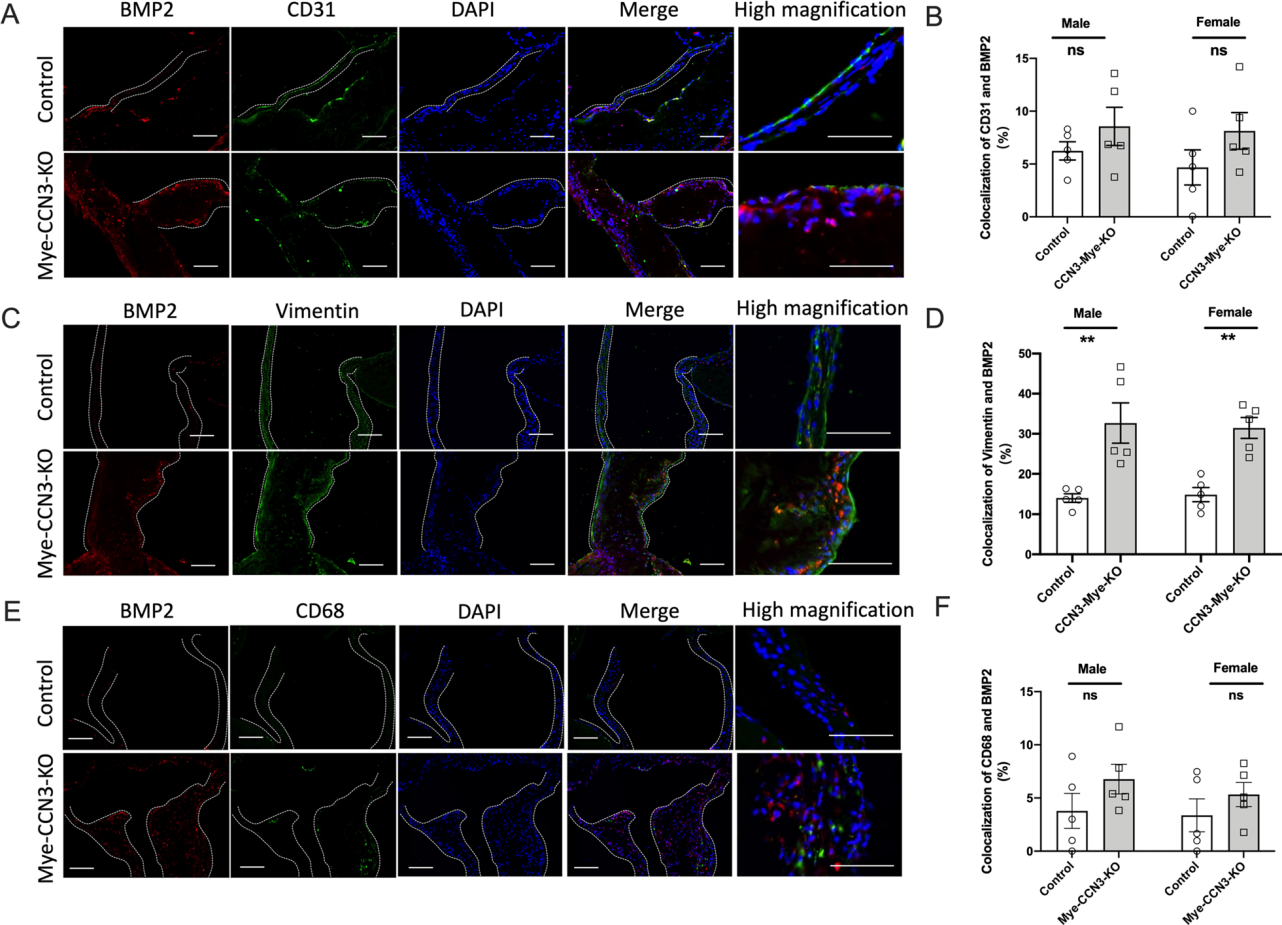
Elevation of BMP2 contributes to the aggravated valvular calcification in the context of myeloid CCN3 deficiency in vivo. **A** Immunofluorescence of BMP2 and CD31 on calcified murine aortic valves. (BMP2: red; CD31: green; DAPI, blue; scale bar: 100 μm, 200 μm). **C** Immunofluorescence of BMP2 and Vimentin on calcified aortic valves. (BMP2: red; Vimentin: green; DAPI, blue; scale bar: 100 μm). **E** Immunofluorescence of BMP2 and CD68 on calcified aortic valves. (BMP2: red; CD68: green; DAPI, blue; scale bar: 100 μm). **B**, **D** and **F** Quantification of percentage of co-stained area of red and green signal on calcified murine aortic valves (n = 5). All Statistical analysis were two-way ANOVA followed by Sidak multiple comparison test. N = biological replicates. Control: Lysm^Cre/Cre^ mice; Mye-CCN3-KO: Myeloid specific CCN3 knock out mice.**p < 0.01

As a secreted protein, macrophage-derived BMP2 may act in a paracrine fashion on series of valvular cell types such as VECs and VICs. To further understand which cell type was mainly affected under myeloid CCN3 deficiencied internal environment on the calcified aortic valves, we performed colocalization immunostaining studies on aortic valves from both control and Mye-CCN3-KO mice from CAVD experiments as described above (Additional file 1: Fig. S1). As illustrated in Fig. 3, quantification of BMP2 colocalization studies revealed no significant increase of BMP2 in VECs (counterstained with CD31) but a strong increase (~ threefold) of BMP2 in VICs was observed in Mye-CCN3-KO mice compared to the controls, indicating that that the upregulation of BMP2 was mostly localized to the VICs and contributed to the calcification of aortic valve leaflets in Mye-CCN3-KO mice. These findings are intriguing because aortic VICs are the critical cell type responsible for valvular remodeling and calcium deposition in the pathogenesis of CAVD [5].

What interesting is immunosaining of CD68 on aortic valves showed a significant increased in Mye-CCN3-KO mice (Additional file 1: Fig. S3A) but colocolization of BMP2 and CD68 in Mye-CCN3-KO mice appeared to be increased but no significant different compared to the controls (Fig. 3E, F). These might be due to more macrophages recruitment happened on the aortic valves after myeloid CCN3 deficiency. Consistently, a significant increase of cell migration was seen in CCN3-deficient macrophages as demonstrated by in vitro scratch assay (Additional file 1: Fig. S3B).And these observations also prompted us to address an important possibility that macrophages possess the capacity to produce and secrete BMP2, and CCN3 deficient macropahges modulates VIC calcification through altering macrophage-produced BMP2, which in turn upregulates Runx2 in VICs to promote calcification.

### CCN3 deficient macrophages emancipated the expression and secretion of BMP2 in vitro

To further confirem the regulatory effect of CCN3 on BMP2 expression in macrophages, primary bone-marrow derived macrophages(BMDM) from Mye-CCN3-KO or control mice were isolated for vitro study. Considering the increased appreciation of macrophage polarization in cardiovascular diseases including CAVD, We first identified the phenotype of these BMDM under basal conditions (without any stimulation). Flow cytometry of MHCII+ and CD206+ were used to assay the gated BMDMs (CD11b+, F4/80+) (Additional file 1: Fig. S4A). There was no difference in MHCII+ and CD206+ cell fractions between the two genotypes. Additionally, at the mRNA level, loss of CCN3 did not impact several key proinflammatory markers such as F4/80, TNFα or IL-10 (Additional file 1: Fig. S4B). These data indicate that CCN3 had minimum influence on macrophage polarization—findings similar to a prior report [33].

**Fig. 4:**
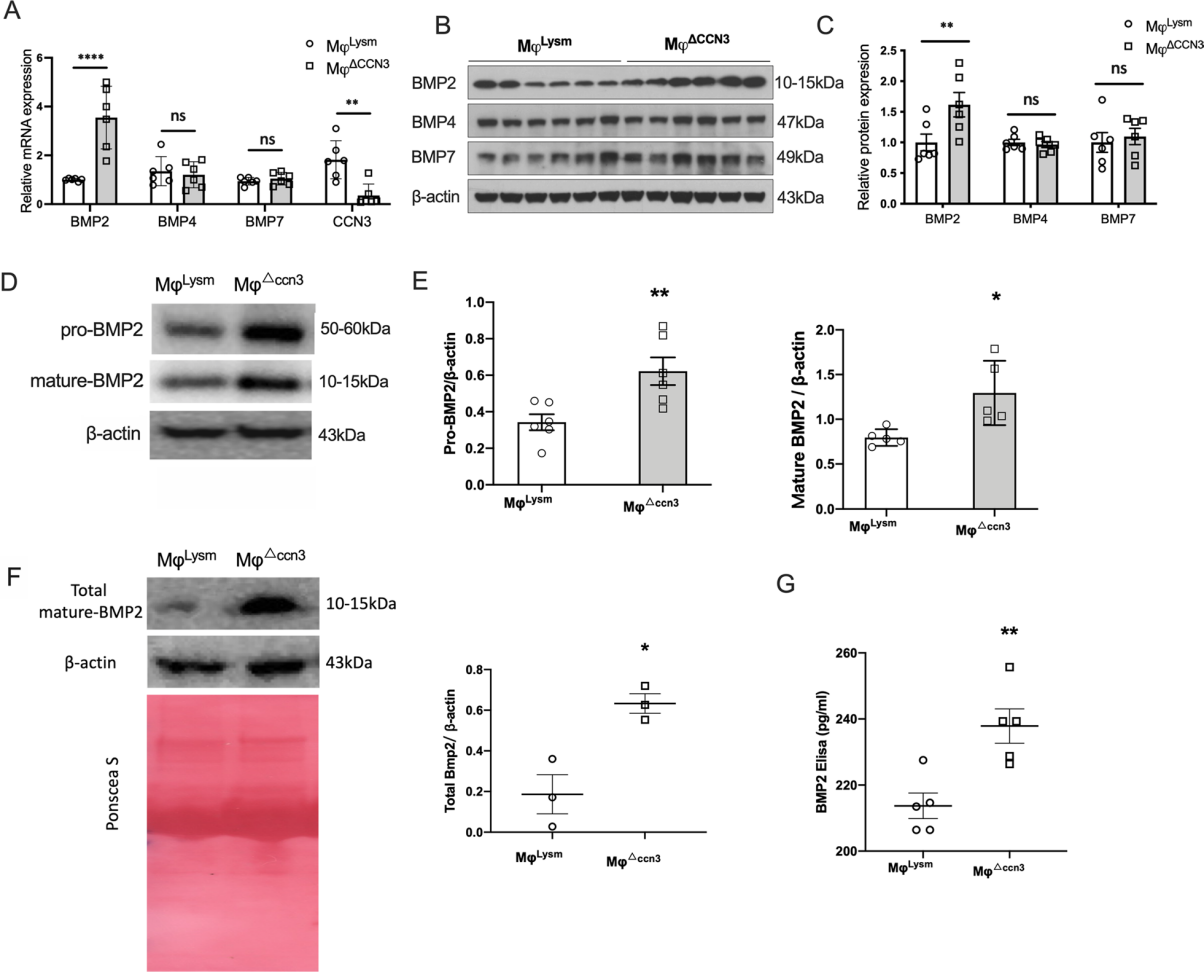
CCN3 deficient macrophages emancipated the expression and secretion of BMP2 in vitro. **A** Relative BMP2, BMP4, BMP7 and CCN3 mRNA expression in bone marrow derived macrophages (n = 6). **B** and **C** Representative immunoblots of BMP2, BMP4 and BMP7 for BMDM lysates from control (Mφ^Lysm^) and CCN3-KO (Mφ^△^.^ccn3^) mice, and the quantification of target proteins were normalized to β-actin. **D** and **E** Representative immunoblots of pro-BMP2, mature-BMP2 for BMDM lysates from control and CCN3-KO mice, and the quantification of target proteins were normalized to β-actin (n = 5). **F** Representative immunoblots of total mature-BMP2 derived from macrophages lysates and cultured medium (n = 3). **G** BMP-2 concentration in macrophage culture supernatant (n = 5). All data analyses were unpaired Student’s *t*-test. N = biological replicates. *p < 0.05. **p < 0.01. ****p < 0.0001

To test our hypothesis that CCN3 serves as an anti-calcifying factor via impacting macrophage behavior and gene expression. We next focused on the mRNA expression of several BMPs in these macrophages. Consistent with our hypothesis, BMP2 mRNA levels were strongly upregulated in Mφ^△ccn3^ while no obvious differences were observed for BMP4 or BMP7, both of which are also related to ossification and able to bind to CCN family members [34, 35] (Fig. 4A). A similar pattern for BMP protein expression was revealed by immunoblots (Fig. 4B, C). Importantly, a strong increase of BMP2 was seen in cell lysate alone (pro- and mature BMP2, Fig. 4D, E), in pooled samples (total mature BMP2 in cell lysate combined with condition medium) (Fig. 4F), and in conditioned medium alone (secreted BMP2, Fig. 4G) derived from Mφ^△ccn3^. Taken together, these data indicate that mature-BMP2 was increased in Mφ^△ccn3^, both intracellularly and extracellularly, suggesting that CCN3 deficiency in macrophages upregulates BMP2 expression, maturation, and secretion.

### Elevation of BMP2 production in CCN3-deficient macrophages propagates VICs calcification in vitro

**Fig. 5:**
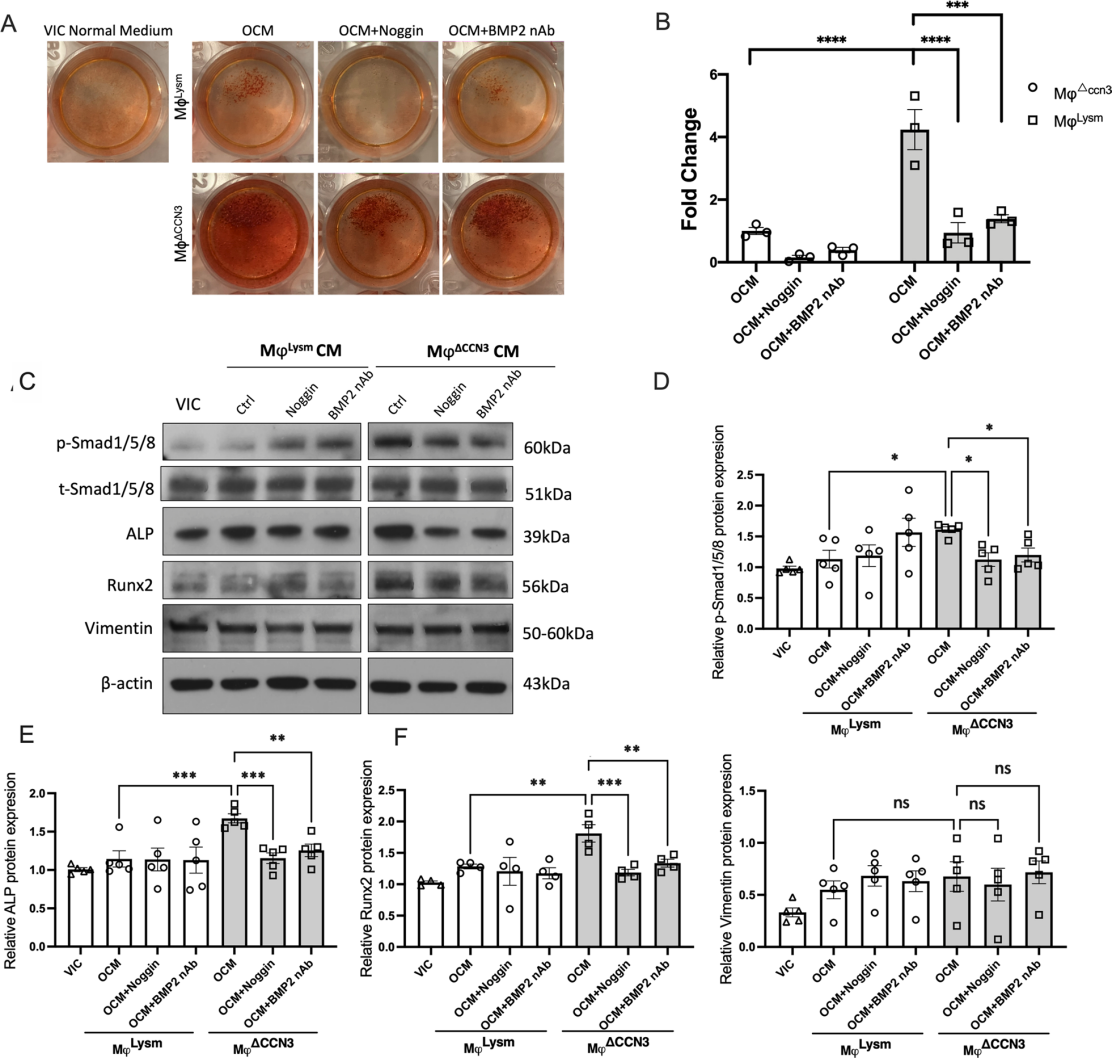
Elevation of BMP2 in CCN3-KO macrophages contributes to the aggravated VICs calcification in vitro. **A** and **B** VICs were co-cultured with OCM for 21 days, Noggin (0.1 μg/ml) and BMP2 nAb (1 μg/ml) were added to block BMP2 activity. Alizarin red staining was performed to reveal matrix mineralization and results quantified with ImageJ (n = 3). **C**–**G** Representative immunoblots of p-SMAD1/5/8, t-SMAD1/5/8, ALP, Runx2 and Vimentin in VICs which had been co-cultured with VIC normal medium, control (Mφ^Lysm^) and CCN3-KO (Mφ^△ccn3^) macrophages conditioned medium (CM) for 7 days (n = 4–5). All Statistical analysis were two-way ANOVA followed by Sidak multiple comparison test. N = biological replicates. Mφ indicates bone marrow derived macrophages; nAb, neutralizing antibody. OCM: Osteogenic conditioned medium.*p < 0.05. **p < 0.01. ***p < 0.001. ****p < 0.0001

To follow up on the above observation and to directly address whether Mφ^△ccn3^ macrophages promote human aortic VICs calcification in vitro, human VICs were cultured in osteogenic media supplemented with Mφ^△ccn3^ conditioned media for 21 days, followed by Alizarin-Red staining. It is clear that a robust calcium deposition (Alizarin red positive area) was seen in VICs cultured with Mφ^△ccn3^ conditioned media (Fig. 5A, B).

While it is evident that Mφ^△ccn3^ secretes more mature BMP2 than Mφ^Lysm^ (BMDMs isolated from control mice) based on our assessment, macrophages produce a variety of cytokines and chemokines that could potentially impact other cells in a paracrine manner. To determine the importance of increased BMP2 secretion from Mφ^△ccn3^ in driving aortic valvular calcification progression, we undertook two different approaches to block BMP2 signaling: (1) utilization of a well-known BMP antagonist–recombinant human Noggin protein, which specifically inhibits the function of several BMPs (like BMP2, BMP4 or BMP7); (2) Adding a BMP2 neutralization antibody [36]. The efficacy of BMP2 inhibition was verified in BMDM transfected with a BMP/Smad Transcriptional Reporter (BRE) construct) (Additional file 1: Fig. S5). Noggin (0.1 μg/ml) or BMP2 neutralizing antibody was added to Mφ^△ccn3^ conditioned medium 1 h prior to its use in VIC culture. Very excitingly, the induction of VIC calcification by Mφ^△ccn3^ conditioned media was markedly suppressed by the inhibition of BMP2 signaling (either via Noggin or BMP2 neutralizing antibody) (Fig. 5A, B).

In a separate set of experiments, we cultured VICs with BMDM conditioned media for seven days, followed by the assessment of key pro-calcifying genes (Runx2 and ALP) and p-Smad1/5/8 (a canonical pathway of BMP2 receptor activation) by western blot. In accordance with the enhancement of calcification seen in VICs cultured with t Mφ^△ccn3^ conditioned media, p-Smad1/5/8, Runx2 and ALP proteins were markedly increased in the VICs cultured in the presence of the Mφ^△ccn3^ conditioned media. Importantly, the addition of Noggin and BMP2 neutralizing antibody potently suppressed the induction of Runx2 and ALP induced by Mφ^△ccn3^ conditioned media. No difference on Vimentin was observed, indicating no impact on VIC proliferation (Fig. 5C–G).

In summary, these data point to the essential role of increased mature BMP2 in the BMDM conditioned media from Mye-CCN3-KO macrophages in the promotion of calcification progression in VICs.

## Discussion

Our recent in vivo studies identified matricellular protein CCN3 as a negative regulator of experimental atherosclerosis. Loss of CCN3 in macrophages results in a marked enhancement of foam cell formation, supporting the potential importance of CCN3 in modulating macrophage biology [16]. Exacerbation of NAFLD as a result of myeloid deficiency of CCN3 provided additional evidence that myeloid CCN3 could impact the behavior of other cell types via a paracrine manner [29]. This study herein provides the first evidence that myeloid deficiency of CCN3 elicited declining valve function and promoted CAVD in mice, thus bolstering the notion that myeloid CCN3 plays a critical role in maintaining tissue homeostasis and is vital for normal cardiovascular physiology.

To explore how macrophage derived CCN3 might limit valvular calcification and CAVD, we analyzed the expression of BMPs in BMDMs. We show that loss of macrophage CCN3 leads to increased expression and secretion of BMP2. This is very intriguing because not only is BMP2 a potent inducer of bone formation through its stimulation of osteoblast differentiation, but more importantly BMP signaling is also required as a critical factor in aortic valve calcification [32, 37]. To confirm that increased BMP2 indeed contributes to VIC calcification, we cultured VICs in osteogenic media supplemented with conditioned media from CCN3-deficient BMDMs. Consistent with the increase Runx2 and ALP expression, a significantly heightened VIC calcification was observed when compared to control conditioned media. Furthermore, inhibition studies using the BMP antagonist Noggin and BMP2 neutralization antibody markedly blunted this effect. Interestingly, CCN3 does not affect BMP4 and BMP7, two other BMP members implicated in vascular calcification [38, 39], indicating the specificity of CCN3 in the regulation of BMP signaling genes. At the mRNA level, we did not observe changes in IL-10 and TNFα (important factors that have a role in valve calcification [40, 41]) (Additional file 1: Fig. S4B). While we cannot exclude the potential contributions from other factors that might be altered due to the loss of CCN3, these data strongly implicate the critical importance of macrophage CCN3 in modulating BMP2 expression and function, and as a result VIC calcification and aortic valve tissue homeostasis. Future studies using open-ended approaches, such as single cell RNA sequencing or secretome proteomics, are required to identify additional molecules responsible for macrophage CCN3’s anti-calcifying effect.

While our data clearly demonstrate that macrophage CCN3 impacts BMP2 production and its downstream functional consequence on VIC calcification biology, there are several noteworthy points to mention about BMP2 expression and action. First, we are aware that loss of myeloid CCN3 might alter the production of BMP2 in cell types other than the macrophages. Our co-staining data indicate that there is heightened BMP2 expression in the VICs but not VECs or macrophages (Fig. 3), further supporting a paracrine effect of macrophage-derived CCN3 on VIC gene expression and function. Second, the regulation of BMP2 signaling by CCN3 requires further investigation. As the largest subfamily of the TGFβ family of growth factors, BMP2 is secreted as a monomer and heterodimerizes with other BMP proteins (ex. BMP2/6, BMP2/7) [42]. Studies have shown that heterodimers can exert more potent activities as compared to their corresponding homodimers [43]. Whether and how CCN3 regulates BMP2 homo or heterodimerization and subsequent receptor engagements, we will be pursued in future studies. Third, the mechanism by which CCN3 regulates BMP2 expression and signaling requires further exploration. Our initial assessment suggests that CCN3 affects BMP2 mRNA, whether this occurs at the transcriptional level or through alteration of RNA stability is not known. It is also possible that CCN3 affects the stability of BMP2 at the protein level. Previous studies have shown that the ubiquitin–proteasome machinery regulates BMP2 and osteoblast differentiation [44, 45]. Of note, CCN1 and CCN4 have been shown to regulate their target gene activity via proteasome-mediated effects on Notch1 and PPAR gamma proteins, respectively [46, 47]. These prior studies raise the intriguing possibility of whether a similar mechanism might be operative for the CCN3-BMP2 axis, an important question that will be explored in our future investigations. A prior in vitro study showed that CCN3 inhibits BMP2-induced osteoblast differentiation by limiting BMP signaling and activation of the Notch pathway [15]. Whether a similar mechanism exists for CCN3 action in macrophages or Notch signaling on VIC merits further study. Taken together, our loss-of-function study strongly supports the beneficial role of myeloid CCN3 in VIC calcification.

In the literature, there have been several studies that have reported somewhat contradicting roles of CCN3 in the regulation of osteoblast differentiation. While most studies describe CCN3 as an inhibitor, a few suggest CCN3 as an inducer of osteoblast differentiation. However, it is noteworthy that most studies that support an inhibitory role of CCN3 are derived from in vivo studies using genetically manipulated animals [48–52]. The results from these studies are in line with our current in vivo observations in Mye-CCN3-KO mice. We also notice that studies based on experiments using recombinant proteins gave inconsistent results. Two studies [53, 54] suggest CCN3 as an inhibitor, while several other studies [34, 55–57] suggest CCN3 as an inducer. It is known that the bioactivity of the mammalian CCN proteins depends upon posttranslational modification; the sources and cell lines used to produce recombinant proteins could drastically impact the interactions of CCN proteins with receptors and other partners and the subsequent biological actions [10]. Also, the cell lines used for producing recombinant proteins and epitope tagging could potentially affect protein activity and function. Therefore, caution should be taken when interpreting data using recombinant proteins produced in *E. coli* for CCN proteins whose bioactivities are regulated by posttranslational modifications. To address whether replenishment of CCN3 could rescue the effects exerted by macrophage ΔCCN3 conditioned medium, future investigations either via genetic reconstitution or using appropriate sources of recombinant proteins that closely mimic the physiological CCN3 are needed.

Previous studies have establishd that sex has implications on the development of different valvular phenotypes, with an increased prevalence of CAVD in male patients. Our in vivo studies did include both sexes, and we did not observe any difference in CAVD between sexes. We recognize one limitation for our in vitro studies were exclusively performed in male BMDMs to gain insight into how macrophage-derived CCN3 affects VIC behavior. There are several considerations for our choice of male macrophages: (1) higher prevalence of CAVD in male patients; (2) avoid any potential female hormonal effects. However, confirming the role of myeloid CCN3 by using cells from both sexes need to be pursued in future investigations.

Our findings here open the venue to specifically address the role of CCN3 derived from diverse cellular sources in valvular calcification and CAVD. While the LysM-Cre line deletes gene expression in the entire myeloid compartment, we initially focused our in vitro studies on macrophages owing to their well-appreciated role in valvular calcification. We are aware that the Cre line (LysM-Cre) utilized in our studies also deletes gene expression in neutrophils. The contribution of neutrophils in valvular calcification and CAVD remains largely underexplored. As the most abundant leukocytes in circulation, neutrophils along with the released neutrophil extracellular traps (NETs) play causal roles in promoting atherosclerosis via multifaceted mechanisms, including altering endothelial and smooth muscle function and platelet activation [58]. Given the undisputed role of neutrophils in atherosclerosis and the presence of calcification in both atherosclerosis and CAVD, it is reasonable to speculate the involvement of neutrophils in valvular calcification and CAVD. A role for neutrophil CCN3 in valvular calcification needs to be explored in future studies involving the use of a murine Cre strain that deletes neutrophil-derived CCN3. Along this same line, knowing the essential importance of the crosstalk between immune cells and valve inhabitant cells (VICs and valvular endothelial cells), it is important to dissect whether and how CCN3 in these cells participates in the maintenance of valve homeostasis. Of note, our previous work shows that atheroprotective flow (laminar shear stress) strongly induces CCN3 in the endothelial cells where it exerts anti-inflammatory actions [59]. Flow-mediated effects in VECs and the functional consequence clearly warrant further investigations. Our preliminary assessment revealed a marked increase of CCN3 protein in human CAVD (Fig. 1A) and co-staining with macrophage marker (CD68) showed a similar pattern. This data, coupled with our findings in animal studies, supports the compensatory action of myeloid CCN3 against CAVD, however further studies are clearly required to address the specific roles of CCN3 in other cell types.

Valvular calcification has been recognized as a diagnostic target of CAVD. Extensive studies currently focus on delineating the fundamental mechanism of CAVD with the goal of identifying novel targets to diagnose and prevent disease progression. In our studies, we perfomed studies in mice with hyperlipidimia (LDLR deficiency) achived via AAV-PCSK9 injection. Sebveral prior studies reported PCSK9 R46L level was associated with CAVD [60–62], and blocking PCSK9 significantly reduced calcium accumulation in human VICs [60]. Additionally, others have demonstrated a single injection of rAAV8/D377Y-mPCSK9 coupled with HFD is a feasible approach to study cardiovascular calcification [20, 63]. Concrodant with prior observations, our study mice developed aortic valve and vascular calcification following AAV-PCSK9 injection and HFD. Furthermore, our results revealed that myeloid CCN3 plays a vital role in limiting valvular calcification and CAVD. Loss of CCN3, by virtue of the subsequent increase of BMP2 release from macrophages and the direct promotion of VIC calcification, contributes to the development of CAVD in mice. Considering the known diverse (opposite on some occasions) and contextual roles of CCN3 in different physiological and pathophysiological conditions, our studies do not imply a potential for exploiting the use of CCN3 for therapy, rather, our observations highlight the novel role of myeloid CCN3 in mitigating CAVD progression.

## Conclusion

To summarize, our data uncovered a novel role of myeloid CCN3 in the regulation of aortic valve calcification. Deficiency of myeloid CCN3 results in the elevation of macrophage secreted BMP2 and the upregulation of a master osteogenic transcription factor Runx2 in VICs, which causes exacerbated VICs calcification, giving rise to valvular dysfunction. In vitro, blocking BMP2 action ablates Mφ△ccn3 conditioned media mediated increase of calcification in VICs. Our results highlight that modulation of BMP2 production and secretion in macrophages might serve as a key mechanism for CCN3’s anti-calcification function in the development of CAVD.

## Supplementary Information

**Additional file 1. Fig. S1**: Murine CAVD model. A. Study protocol for CAVD in mice. B. Representative images of aortic valvular regurgitation detected by Spectral Doppler in mice. An entire peak of regurgitation was defined as severe regurgitation (upper panel), and a partial peak was defined as mild regurgitation (lower panel). C. Distribution of aortic valve regurgitation in control and Mye-CCN3-KO mice (n=16, 8 males and 8 females). Control: Lysm^Cre/Cre^ mice; Mye-CCN3-KO: Myeloid specific CCN3 knock out mice. **Fig. S2**: Plasma lipid profile of mice post 40 weeks of high fat diet feeding. All Statistical analysis were two-way ANOVA followed by Sidak multiple comparison test. N=biological replicates. CHOL indicates cholesterol; TG, Triglyceride; HDLC: High density lipoprotein cholesterol; none HDLc: Non-high-density lipoprotein cholesterol; LDLc, Low-Density Lipoprotein Cholesterol; Control: Lysm^Cre/Cre^ mice; Mye-CCN3-KO: Myeloid specific CCN3 knock out mice. **Fig. S3**: Loss of CCN3 causes an increase of macrophages migration in vivo and vitro. A. Representative immunostaining for CD68 on aortic valve of Control and Mye-CCN3-KO mice. Scale bar=100μm. Quantification of the relative fold change of positive stained area to the entire valve leaflet area in comprision to the control group (n=5). B. Scratch assay for primary macrophages cultured in DMEM. Images were analyzed with ImageJ. Scale bar: 200μm; n=6. Data analysis were unpaired Student’s t-test. N=biological replicates. Control: Lysm^Cre/Cre^ mice; Mye-CCN3-KO: Myeloid specific CCN3 knock out mice; Mφ^lysm^: BMDMs from Control mice; Mφ^△CCN3^: BMDMs from Mye-CCN3-KO mice. *p<0.05. **p<0.01. **Fig. S4**: Characterization of bone marrow-derived macrophages. A. Characterization of primary murine macrophages by flow cytometry. Percentage of MHC II+ and CD206+ within CD11b+ and F4/80+ controls (n=5). B. Relative CCN3, F4/80, IL-10 and TNFα expression in BMDMs (n=6). All data analyses were unpaired Student’s t-test. N=biological replicates. Mφ^lysm^: BMDMs from Control mice; Mφ△CCN3: BMDMs from Mye-CCN3-KO mice. ****p<0.0001. **Fig. S5**: Verification of the blocking efficacy of BMP2 nAb in vitro. A. Diagram for verification of BMP2 nAb *in vitro*. B. BMDMs were transfected with pGL3 luciferase plasmid. After 24 hours, cells were treated with BMP2 (10 ng/ml) in the presence or absence of BMP2 nAb (1μg/ml) for 24 hours prior to measuring luciferase activity (n=4). *p<0.05.

## Data Availability

All data in our study are available upon request.

## Abbreviations

AVICs: Human aortic valve interstitial cells
BMP2: Bone morphogenetic protein 2
BMDMs: Bone marrow-derived macrophages
CAVD: Calcific aortic valve disease
CCN: Cellular communication network
CCN3: Cellular communication network factor 3
DMEM: Dulbecco's Modified Eagles medium
ECM: Extracellular matrix
EF: Ejection fraction
FGF2: Fibroblast growth factor 2
HFD: High fat diet
LDLR: Low density lipoprotein receptor
LV: Left ventricle
Mφ^△^^ccn3^ & Mφ^Lysm^: BMDMs isolated from Mye-CCN3-KO mice or controls
Mye-CCN3-KO: Myeloid-specific CCN3 knockout
NAFLD: Non-alcoholic fatty liver disease
NETs: Neutrophil extracellular traps
VICs: Valvular interstitial cells
VECs: Valve endothelial cells
VTI: Velocity time integral
