# Supplementary figures and images for: Myeloid CCN3 protects against aortic valve calcification
Source: Cell Commun Signal. 2023 Jan 20;21:14. doi: 10.1186/s12964-022-01020-0 (PMC9854076; doi:10.1186/s12964-022-01020-0)

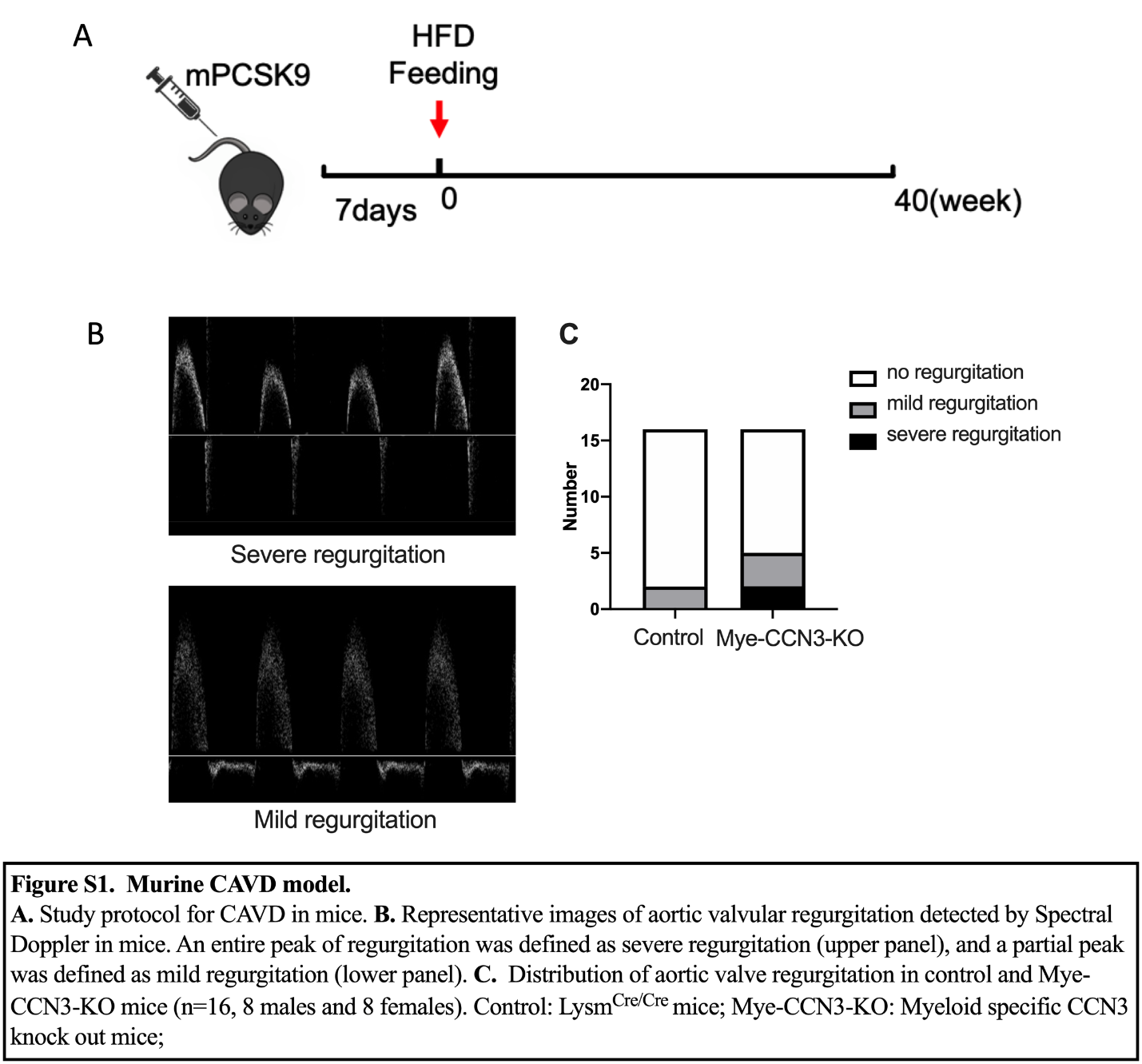
SUPPLEMENTAL MATERIAL
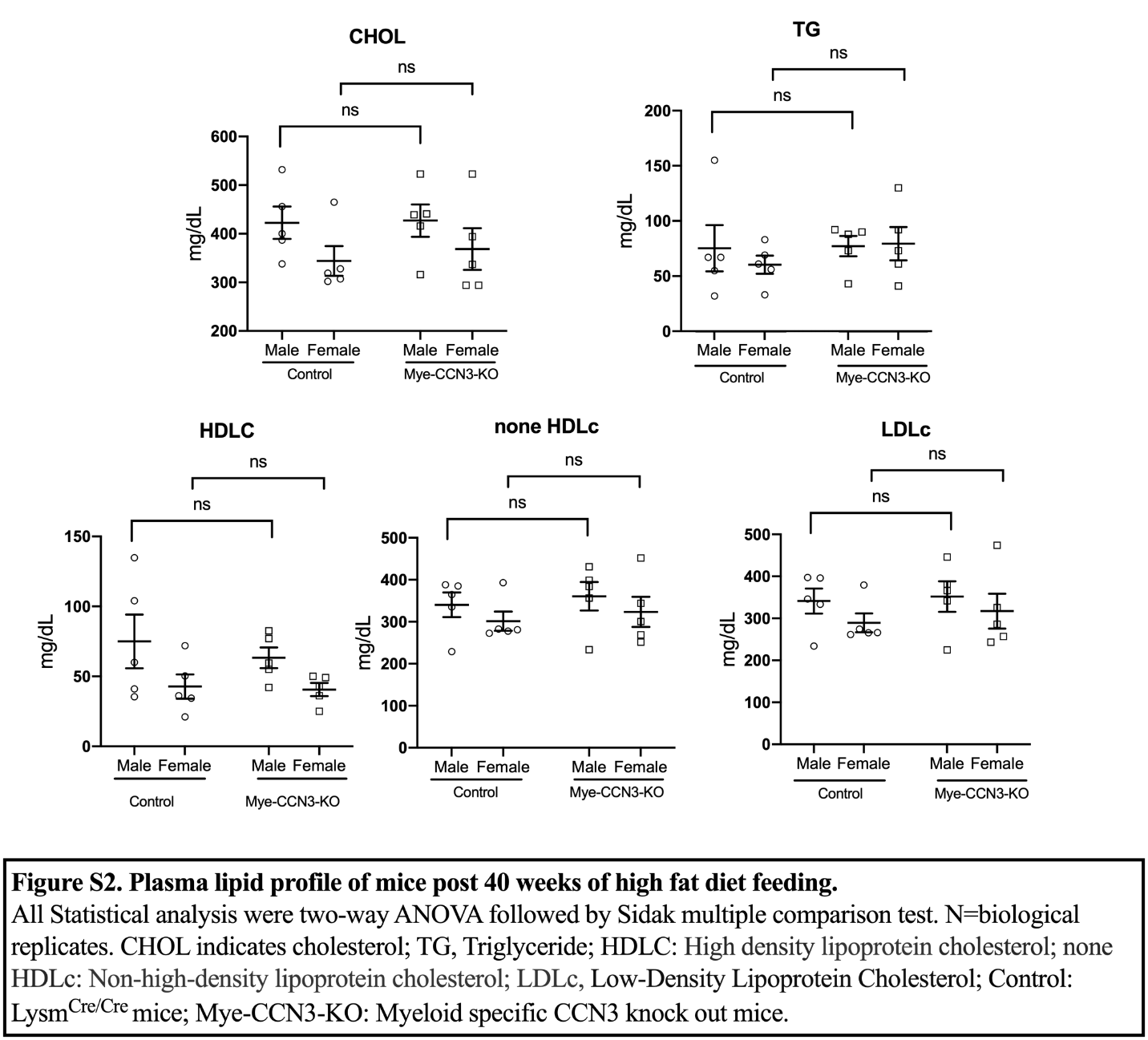

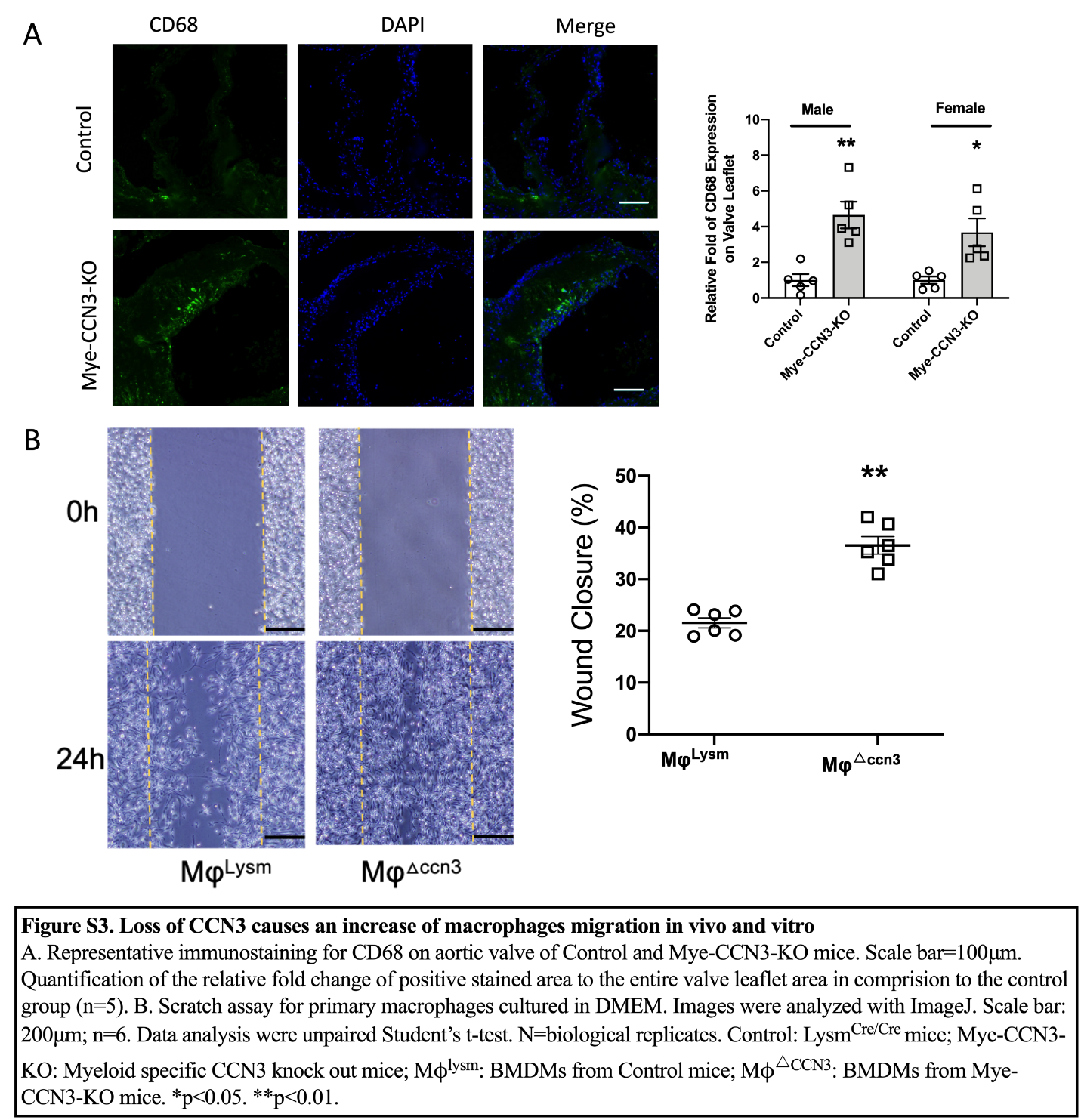

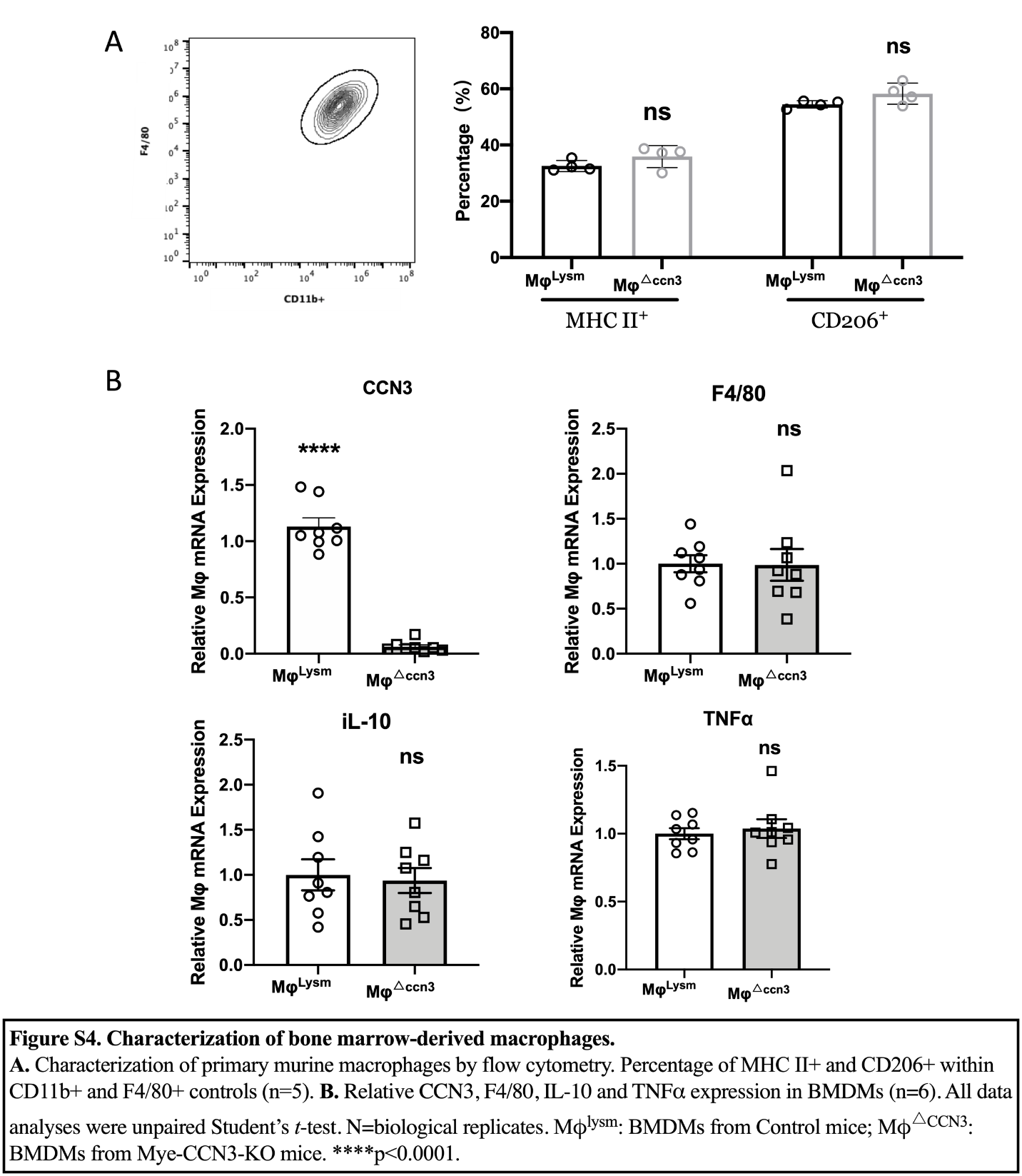

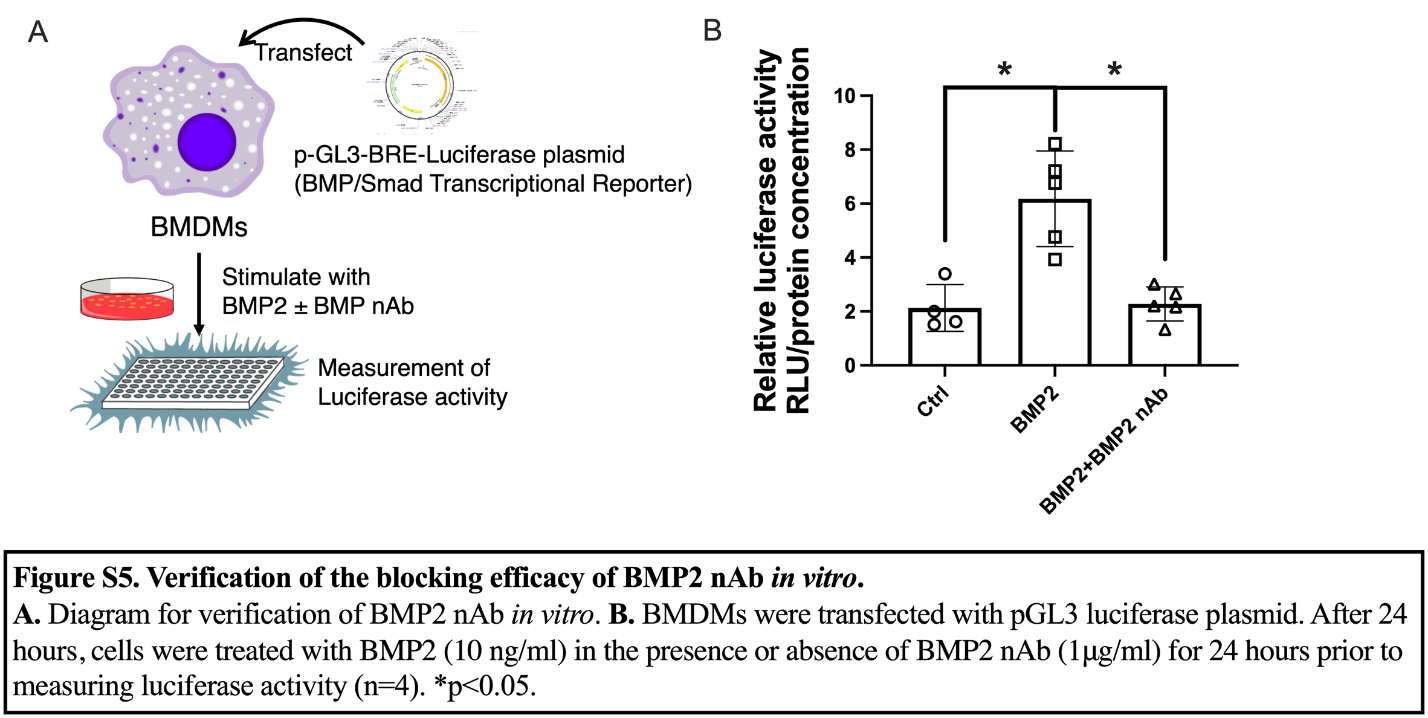

Supplement: Supplementary file 2 — Additional file 1. Fig. S1: Murine CAVD model. A. Study protocol for CAVD in mice. B. Representative images of aortic valvular regurgitation detected by Spectral Doppler in mice. An entire peak of regurgitation was defined as severe regurgitation (upper panel), and a partial peak was defined as mild regurgitation (lower panel). C. Distribution of aortic valve regurgitation in control and Mye-CCN3-KO mice (n=16, 8 males and 8 females). Control: LysmCre/Cre mice; Mye-CCN3-KO: Myeloid specific CCN3 knock out mice. Fig. S2: Plasma lipid profile of mice post 40 weeks of high fat diet feeding. All Statistical analysis were two-way ANOVA followed by Sidak multiple comparison test. N=biological replicates. CHOL indicates cholesterol; TG, Triglyceride; HDLC: High density lipoprotein cholesterol; none HDLc: Non-high-density lipoprotein cholesterol; LDLc, Low-Density Lipoprotein Cholesterol; Control: LysmCre/Cre mice; Mye-CCN3-KO: Myeloid specific CCN3 knock out mice. Fig. S3: Loss of CCN3 causes an increase of macrophages migration in vivo and vitro. A. Representative immunostaining for CD68 on aortic valve of Control and Mye-CCN3-KO mice. Scale bar=100μm. Quantification of the relative fold change of positive stained area to the entire valve leaflet area in comprision to the control group (n=5). B. Scratch assay for primary macrophages cultured in DMEM. Images were analyzed with ImageJ. Scale bar: 200μm; n=6. Data analysis were unpaired Student’s t-test. N=biological replicates. Control: LysmCre/Cre mice; Mye-CCN3-KO: Myeloid specific CCN3 knock out mice; Mφlysm: BMDMs from Control mice; Mφ△CCN3: BMDMs from Mye-CCN3-KO mice. *p<0.05. **p<0.01. Fig. S4: Characterization of bone marrow-derived macrophages. A. Characterization of primary murine macrophages by flow cytometry. Percentage of MHC II+ and CD206+ within CD11b+ and F4/80+ controls (n=5). B. Relative CCN3, F4/80, IL-10 and TNFα expression in BMDMs (n=6). All data analyses were unpaired Student’s t-test. N=biological re [file 12964_2022_1020_MOESM2_ESM.docx]
